# Construction of nomogram based on clinical factors for the risk prediction of postoperative complications in children with choledochal cyst

**DOI:** 10.3389/fped.2024.1372514

**Published:** 2024-08-07

**Authors:** Yang Lin, Xinru Xu, Shan Chen, Ling Zhang, Jianbin Wang, Xinyi Qiu, Lizhi Li

**Affiliations:** ^1^ Department of Pediatric Surgery, Provincial Clinical Medical College, Fujian Medical University, Fuzhou, Fujian, China; ^2^ Department of Hematology, Provincial Clinical Medical College, Fujian Medical University, Fuzhou, Fujian, China

**Keywords:** choledochal cyst, pediatric, postoperative complications, nomogram, logistic regression

## Abstract

**Objective:**

The aim of the study was to develop a prediction nomogram based on clinical factors to assess the risk of postoperative complications in children with congenital choledochal cyst.

**Methods:**

The clinical data from 131 children who underwent choledochal cyst resection and Roux-en-Y hepaticojejunostomy in our hospital between January 2016 and December 2022 were retrospectively analyzed. The general information, clinical symptoms, procedure, biochemical indicators, and imaging data were recorded. A prolonged hospital stay induced by postoperative complications or a follow-up over 6 months was assessed as the event outcome. A logistics regression analysis was performed to screen for risk factors with statistical significance in inducing postoperative complications. Then, with the dataset split into the training group and internal validation group, the nomogram for the prediction of postoperative complications was developed based on a computer algorithm. In addition, the receiver operating characteristic (ROC) curve and calibration curve were performed for nomogram verification.

**Results:**

Of 131 children, the multivariate logistics regression analysis suggested that age ≤2 years [odds ratio (OR) 0.93; 95% confidence interval (CI) 0.15–5.65; *p *= 0.938], Todani classification type 1 (OR 36.58; 95% CI 4.14–871.74; *p *= 0.005), cyst wall thickness >0.4 cm (OR 10.82; 95% CI 2.88–49.13; *p < *0.001), with chronic cholecystitis (OR 7.01; 95% CI 1.62–38.52; *p *= 0.014), and choledochal cyst diameter (OR 1.01; 95% CI 0.99–1.03; *p *= 0.370) were predictors associated with the postoperative complications of choledochal cysts. The data were randomly divided into the training group (*n* = 92) and internal validation group (*n* = 39) to build the prediction nomogram including the appeal factors. The accuracy and discrimination of the model were evaluated using a ROC curve and calibration curve. The results showed that the nomogram area under the ROC curve [area under the curve (AUC) = 0.894; 95% CI 0.822–0.966; *p *< 0.001], validation (AUC = 0.844; 95% CI 0.804–0.952; *p *< 0.001), and Brier = 0.120 (95% CI 0.077–0.163p; *p *< 0.001) were indicative of the good stability and calibration of the predictive nomogram.

**Conclusion:**

The prognosis of congenital choledochal cysts was associated with multiple aspects of clinical factors. Combined with the internal validation, the novel prediction nomogram was suitable for evaluating the individualized risk of postoperative complications of choledochal cysts. The prediction nomogram could provide a more accurate strategy of procedure and postoperative follow-up for children with choledochal cysts.

## 1 Introduction

The choledochal cyst, also known as biliary dilatation (BD), is a rare congenital anomaly characterized by dilation of the intrahepatic and extrahepatic biliary tree (1). It is predominantly seen in Asians, with an incidence of approximately 0.1%. It is more common in girls and presents with symptoms such as recurrent abdominal pain, painless jaundice, and abdominal masses (2, 3). The condition is classified into five types according to the Todani classification (4), with types I and IV being the most prevalent (5). Type I encompasses cystic dilatation (Ia) and fusiform dilatation (Ic), and is often associated with pancreaticobiliary maljunction. Type IV includes multiple intrahepatic and extrahepatic cysts (IVa) and multiple extrahepatic biliary cysts (IVb).

The etiological mechanism of choledochal cyst mainly includes two theories: pancreaticobiliary maljunction and congenital biliary stricture (6, 7). A choledochal cyst could induce progressive hepatic failure, biliary perforation, and even carcinogenesis, as well as clinical complications resulting from pancreaticobiliary maljunction (1, 8). For patients with choledochal cysts, radical excision of the cyst is more efficient than internal drainage surgery, preventing biliary tumorigenesis and hepaticojejunostomy anastomotic stricture (9, 10). Nowadays, the mainstream procedure for choledochal cysts is radical resection of the cyst, gallbladder, and dilated cholangio, followed by reconstruction of the biliary tract and pancreatobiliary shunt via Roux-en-Y hepaticojejunostomy (11).

Laparoscopic choledochal cyst excision (LCCE) and robot-assisted laparoscopic choledochal cyst excision (RALCCE) with the Roux-en-Y hepaticojejunostomy has been widely applied to the treatment of children with choledochal cysts (12, 13). However, limited surgical visualization, anatomical anomalies of the hepatobiliary duct, and cyst wall inflammation in pediatric patients increase procedural difficulty (14, 15). In addition, post-choledochal cyst excision complications, such as biliary fistula recurrence, pancreatitis, and biliary stricture, are associated with newborn patients, pancreaticobiliary maljunction, Todani classification, and lesion inflammation (16–19). Therefore, it is essential to identify risk factors to guide efficient therapy and perioperative care for children with choledochal cysts.

The clinical regression nomogram is utilized for disease diagnosis, therapy formulation, and prognosis survival analysis. It has been applied for risk assessment in choledochal cyst carcinogenesis (20) as well as surgical risk prediction in pancreaticobiliary maljunction and bile duct dilatation (21). However, there is a research gap in postoperative complications of choledochal cysts in children, and no clinical prediction nomogram for prognosis exists. Therefore, the aim of our research was to establish a regression nomogram based on the perioperative data of children with choledochal cysts. We will identify risk variables and develop a generalized nomogram to assess the incidence of complications of Roux-en-Y hepatoenteric anastomosis, aiming to enhance procedural efficiency and prognosis in children with choledochal cysts.

## 2 Materials and methods

### 2.1 Clinical information

**Figure 1:**
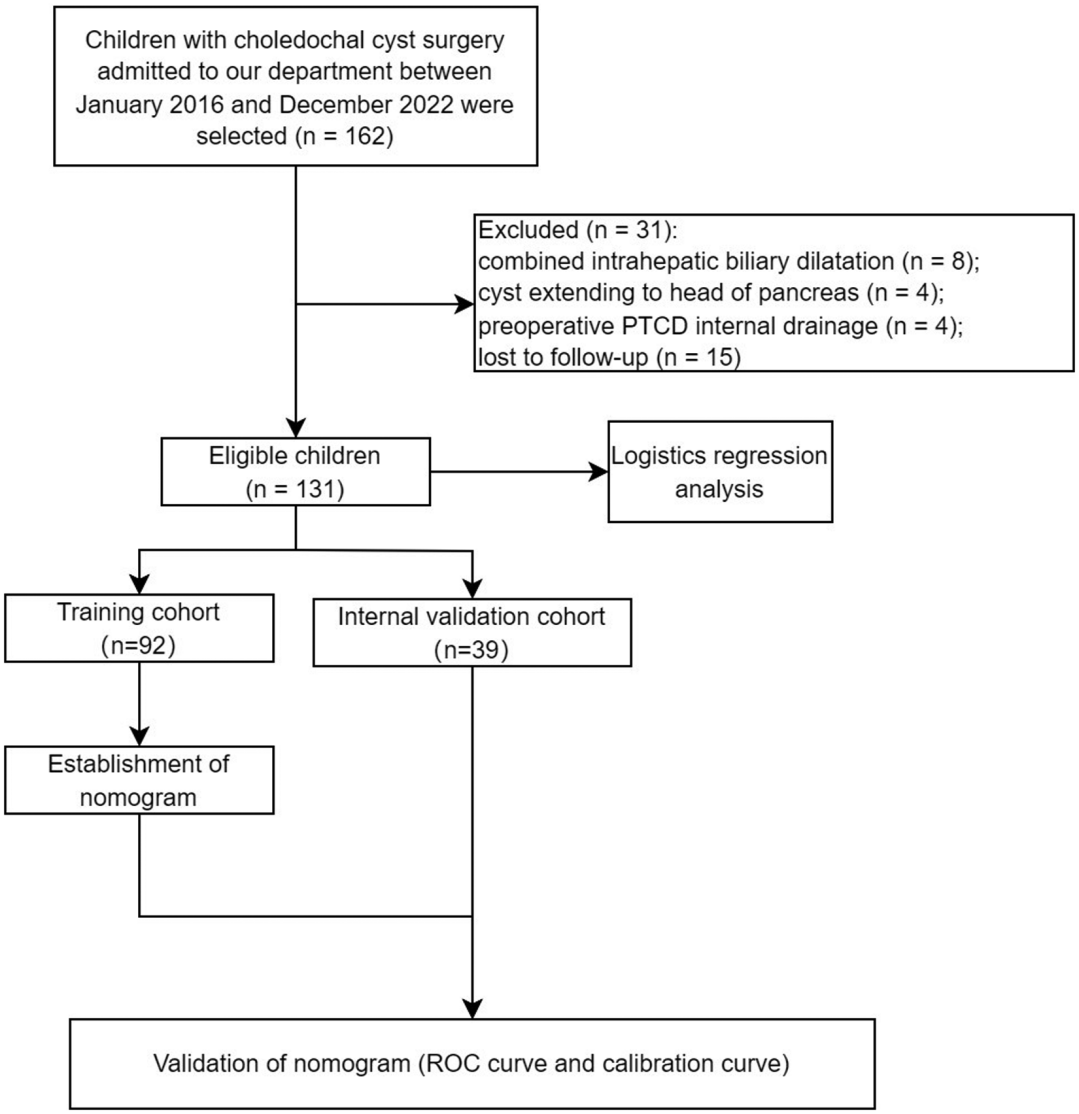
The process of data of children with choledochal cyst.

The data of 131 children who underwent surgical treatment for choledochal cysts in our department between January 2016 and December 2022 were retrospectively analyzed (Figure 1). Both laparoscopic and robotic-assisted laparoscopic Roux-en-Y hepaticojejunostomy were completed by one field organization. Based on the postoperative follow-up ≥6 months, the research endpoint was dependent on whether there was an occurrence of defined complications or not.

The inclusion criteria were as follows: (1) patients with a cholangiectasis diameter ≥10 cm; (2) patients experiencing clinical symptoms, or pancreaticobiliary maljunction, with a biliary diameter <10 mm; (3) patients whose parents agreed and signed the informed consent; and (4) patients with complete data of clinical case, laboratory tests, and imaging results. The exclusion criteria were as follows: (1) patients with intrahepatic biliary dilatation, requiring a radical resection of the liver lesion; (2) choledochal cysts with pancreatic infiltration; (3) patients undergoing percutaneous transhepatic cholangio drainage (PTCD) or other catheter drainage preoperatively; (4) patients with tumorigenesis of the cyst or hepatobiliary tube; and (5) patients intolerant of surgery.

### 2.2 The acquiring of perioperative data

The potential risk factors associated with the prognosis of children with choledochal cysts were recorded and included in the study. The following independent variables were included: sex; age; clinical symptoms; surgical procedures; cyst diameter; Todani classification; cyst wall thickness; occurrence of chronic cholangitis; and the level of direct bilirubin and serum amylase. The postoperative recovery of the children was assessed with a median follow-up of 48 months (range 6–76). Postoperative complications were used as an assessment index, including the mid- to long-term complications confined to anastomotic leakage, cholangitis, pancreatitis, or stenosis of the anastomotic site. During the follow-up, there were 25 patients suffering from the defined complications leading to a prolongation of hospital stay, with an incidence of 19.08%.

The postoperative complications were defined as follows:

(1) Mid- to long-term anastomotic leakage: often diagnosed clinically by closely monitoring drainage volume, abdominal signs, and infection indicators, as there may be subtle or tolerant abdominal pain, often with encapsulated fluid in the hepatic region on ultrasound.
(2) Stenosis of the anastomotic site: abdominal pain with jaundice aggravation or unexplained intermittent abdominal pain, repeated low-grade fever, and increased transaminases. The imaging examination shows that the bile duct or intrahepatic bile duct above the obstruction point is dilated or not dilated.
(3) Cholangitis: abdominal pain with jaundice aggravation not resulting from anastomotic stenosis.
(4) Pancreatitis: abdominal pain with serum amylase aggravation.

### 2.3 Risk factor analysis and construction of clinical prediction model

Taking the occurrence of postoperative complications of choledochal cysts as the event outcome (with postoperative complications inducing a prolongation of hospital stay or without), sex (boy or girl), age (≤2 years or >2 years), clinical symptoms (with or without), procedure (LCCE, RALCCE), cyst classification (type I or IVb), thickness of cyst wall (>0.4 or ≤0.4 cm), chronic cholecystitis (with or without), cyst diameter (cm), serum amylase (U/L), and direct bilirubin (μmol/L) were included in the equation for univariate logistic regression analysis and further screening of factors. Of children (Table 1), algorithm 92 cases were randomly selected as the training cohort, inclusion of males (*n* = 25) and females (*n* = 67); and type I (*n* = 44) and type IV (*n* = 48). There were 44 cases undergoing the laparoscopic Roux-en-Y hepaticojejunostomy, of which 12 cases suffered from defined complications and 32 cases recovery well; and 48 cases undergoing robot-assisted Roux-en-Y hepaticojejunostomy, of which 7 cases suffered from defined complications and 41 cases recovery well. The property and discrimination of the prediction nomogram were statistically assessed by internal validation, the area under the receiver operating characteristic (ROC) curve (AUC) and calibration curve.

**Table 1:** Patient demographics and baseline characteristics.

Characteristic	Cohort		*p-*value
	Training cohort *N* = 92	Internal test cohort *N* = 39	
Gender, *N* (%)			0.904
Girl	67 (72.8%)	28 (71.8%)	
Boy	25 (27.2%)	11 (28.2%)	
Age (years), *N* (%)			0.420
>2	52 (56.5%)	25 (64.1%)	
≤2	40 (43.5%)	14 (35.9%)	
Clinical symptoms, *N* (%)			0.998
Without	59 (64.1%)	25 (64.1%)	
With	33 (35.9%)	14 (35.9%)	
Procedure, *N* (%)			0.243
RALCCE	48 (52.2%)	16 (41.0%)	
LCCE	44 (47.8%)	23 (59.0%)	
Todani classification, *N* (%)			0.252
Type Ⅳb	49 (53.3%)	25 (64.1%)	
Type Ⅰ	43 (46.7%)	14 (35.9%)	
Thickness of cyst wall (cm), *N* (%)			0.422
≤0.4	67 (72.8%)	31 (79.5%)	
>0.4	25 (27.2%)	8 (20.5%)	
Diameter of cyst (cm)			0.444
Median (IQR)	3.35 (2.17–5.25)	3.10 (2.00–4.50)	
Chronic cholecystitis, *N* (%)			0.063
Without	50 (54.3%)	28 (71.8%)	
With	42 (45.7%)	11 (28.2%)	
Serum amylase (U/L)			0.708
Median (IQR)	56 (49–80)	59 (56–79)	
Direct bilirubin (μmol/L)			0.571
Median (IQR)	3 (1–7)	2 (1–5)	

### 2.4 Common preoperative preparations

The preoperative bowel lavage and antibiotic prophylaxis were necessary for routine bowel preparations before surgery. After the indwelling of the gastric canal and urinary catheter and under general anesthesia, the patient was placed in a reverse-Trendelenburg position. The patient's bed was tilted to the left by 30°–45° after fixation with the elevated abdominal lesion.

### 2.5 The robotic procedure and laparoscopic devices

The devices used were as follows: (1) the Da Vinci robotic control system (Da Vinci, Mountain View, CA, USA); (2) the single-port multichannel trocar in robotic surgery and traditional trocar in laparoscopy (Sunride, Changzhou, Jiangsu, China); and (3) SRORZ pediatric laparoscopic devices (SYZBA-2010142, 7220BA/26005BAK).

### 2.6 The robotic Roux-en-Y hepaticojejunostomy procedure

The extracorporeal jejunostomy involved the exploration of the abdominal cavity via laparoscopy; a puncture decompression was performed first to solve the obvious dilation of the common bile duct (CBD) and gallbladder. Then, the jejunum, 25 cm away from the ligament of Treitz, was clamped and stretched extracorporeally. After cutting the jejunum, a 4 cm overlap of lateral anastomosis was performed between the proximal jejunum and the distal jejunum 20 cm away from the canaliculus breakage. Ensuring no fistula in the anastomosis for patency, the mesangial hiatus was sutured and closed. The lateral wall of the mesangial margin was incised on both sides of the folded jejunum for a sixth gastric tube to suck out the duodenal juice; and the jejunal end was temporarily closed with 5-0 Vicryl sutures for a subsequent hepaticojejunostomy.

A single-port plus-one approach was performed for robotic procedure after the establishment of pneumoperitoneum (6–10 mmHg), and the Xi robotic system devices were anchored: a single-port multichannel trocar was placed into an arc incision of 25–30 mm around the umbilicus, with the anchoring of camera arm Ⅲ and operation arm 2163;. Operation arm Ⅱ was anchored into an 8 mm plus-one port 6 cm away from the right flat umbilicus.

The gallbladder and ligamentum teres hepatis were suspended by 4-0 absorbable sutures passing through the right costal margin and subxiphoid abdominal wall to fully expose the hilum. The mesentery was incised to enable the jejunal loop of the hepatic branch carried to the hilum through the retrocolic tunnel and fixed with the appropriate length. The gallbladder was detached from the CBD along the submucosa of the gallbladder fundus and removed. The anterior wall of the cyst was incised, and the cyst was transected after mobilization along the posterior wall of the cyst. The distal cyst was detached from the pancreaticobiliary junction, the distal end of CBD was clipped and the distal cyst was resected; the proximal cyst was also removed until the CBD was non-expansive. The common hepatic duct was suspended and continuously sutured with the jejunal loop of the hepatic branch. After abdominal exploration, the abdominal cavity was closed methodically.

### 2.7 The laparoscopic Roux-en-Y hepaticojejunostomy procedure

The traditional quadripuntal approach was performed laparoscopically: a 12 mm trocar was placed under the umbilicus as lens port 3, and a 5 mm trocar was placed at a distance of 6 cm from the left and right sides of the umbilicus as operation ports 2 and 4, respectively; a 5 mm trocar was placed between the lens port and operation port 2 as auxiliary port 5. The excision of the choledochal cyst and gallbladder was performed after anchoring the laparoscope. Subsequently, the anastomosis of the overlapping jejunum was completed outside the abdomen. Then, the Roux-en-Y hepaticojejunostomy was finished laparoscopically (22).

### 2.8 Statistical analysis

SPSS version 23.0 software (IBM Corp., Armonk, NY, USA) was used for the data analysis. Qualitative data were represented as *n* (%), and the *χ*^2^ test was performed for the between-group comparison. The quantitative data were analyzed using the Shapiro–Wilk test for normality of data. The mean ± standard deviation was used to describe the continuous variables with normality, the paired-samples *t*-test was used for the between-group comparison, and Pearson's test was used to describe the linear correlation. Median (*Q*1, *Q*3) was used to describe continuous variables with the abnormality, and the Wilcoxon rank-sum test was used for comparison between groups. Univariate and multivariate logistic regression were used for the analysis of risk factors (*p *< 0.05). R software 4.2.2 and MSTATA were used to randomly sample into the training and internal validation cohorts with a ratio of 2:1, and further screening factors for the construction of the predictive nomogram. The AUC and calibration curve were also computed using the R package to evaluate the discriminant validity and predictive ability of the nomogram.

### 2.9 Ethical statement

The study was approved by the Fujian Province Hospital ethics committee (Ethics Approval Number: 2018-LB-066). In addition, all the necessary informed consent was obtained from the legal guardians of all patients before researching the relevant procedures.

## 3 Results

### 3.1 Risk factors for postoperative complications of choledochal cysts based on logistic regression analysis

The results of logistic univariate regression showed that being male [odds ratio (OR) 1.31; 95% confidence interval (CI) 0.49–3.30; *p *= 0.574], aged ≤2 years (OR 11.61; 95% CI 4.05–42.23; *p *< 0.001), with clinical symptoms (OR 1.87; 95% CI 0.77–4.55; *p *= 0.164), LCCE (OR 2.38; 95% CI 0.97–6.28; *p *= 0.066), Todani classification type I (OR 53.09; 95% CI 10.53–969.15; *p *< 0.001), cyst wall thickness >0.4 cm (OR 11.95; 95% CI 4.56–33.92; *p *< 0.001), with chronic cholecystitis (OR 12.14; 95% CI 4.22–44.20; *p *< 0.001), choledochal cyst diameter (OR 1.39; 95% CI 1.15–1.73; *p *= 0.055), and direct bilirubin level (OR 1.03; 95% CI 1.02–1.06; *p *= 0.001) were risk factors for postoperative complications of congenital choledochal cysts. The results of the logistic multivariate regression showed that cyst wall thickness >0.4 cm (OR 10.82; 95% CI 2.88–49.13; *p < *0.001), Todani classification type I (OR 36.58; 95% CI 4.14–871.74; *p *= 0.005), and chronic cholecystitis (OR 7.01; 95% CI 1.62–38.52; *p *= 0.014) were risk factors for postoperative complications of congenital choledochal cysts (*p *< 0.05) (Table 2).

**Table 2:** Univariate and multivariate analysis of influencing factors (logistic regression).

Dependent: Postoperative. complications	Non-event	Event	OR (univariable)	OR (multivariable)
Gender, *N* (%)				
Girl	95 (84.8)	17 (15.2)	—	—
Boy	36 (81.8)	8 (18.2)	1.31 (0.49–3.30, *p* = 0.574)	—
Age (years), *N* (%)				
>2	77 (95.1)	4 (4.9)	—	—
≤2	54 (72.0)	21 (28.0)	11.61 (4.05–42.23, *p* < 0.001)	0.93 (0.15–5.65, *p* = 0.938)
Clinical symptoms, *N* (%)				
Without	84 (86.6)	13 (13.4)	—	—
With	47 (79.7)	12 (20.3)	1.87 (0.77–4.55, *p* = 0.164)	—
Procedure, *N* (%)				
RALCCE	64 (88.9)	8 (11.1)	—	—
LCCE	67 (79.8)	17 (20.2)	2.38 (0.97–6.28, *p* = 0.066)	—
Thickness of cyst wall (cm), *N* (%)				
≤0.4	98 (92.5)	8 (7.5)	—	—
>0.4	33 (66.0)	17 (34.0)	11.95 (4.56–33.92, *p* < 0.001)	10.82 (2.88–49.13, *p* < 0.001)
Todani classification, *N* (%)				
Type Ⅳb	74 (98.7)	1 (1.3)	—	—
Type Ⅰ	57 (70.4)	24 (29.6)	53.09 (10.53–969.15, *p* < 0.001)	36.58 (4.14–871.74, *p* = 0.005)
Chronic cholecystitis, *N* (%)				
Without	78 (95.1)	4 (4.9)	—	—
With	53 (71.6)	21 (28.4)	12.14 (4.22–44.20, *p* < 0.001)	7.01 (1.62–38.52, *p* = 0.014)
Diameter of cyst (cm)				
Median (Q1, Q3)	3.05 (2.00, 4.10)	3.68 (2.50, 5.25)	1.39 (1.15–1.73, *p* = 0.055)	—
Direct bilirubin (μmol/L)				
Median (Q1, Q3)	2 (1, 5)	3 (2, 8)	1.03 (1.02–1.06, *p* = 0.001)	1.01 (0.99–1.03, *p* = 0.370)
Serum amylase (U/L)				
Median (Q1, Q3)	53 (49, 72)	59 (55, 85)	1.00 (0.99–1.00, *p* = 0.569)	—

### 3.2 Nomogram construction of risk factors for postoperative complications of choledochal cysts

#### 3.2.1 Variable selection and nomogram estimation

**Figure 2:**
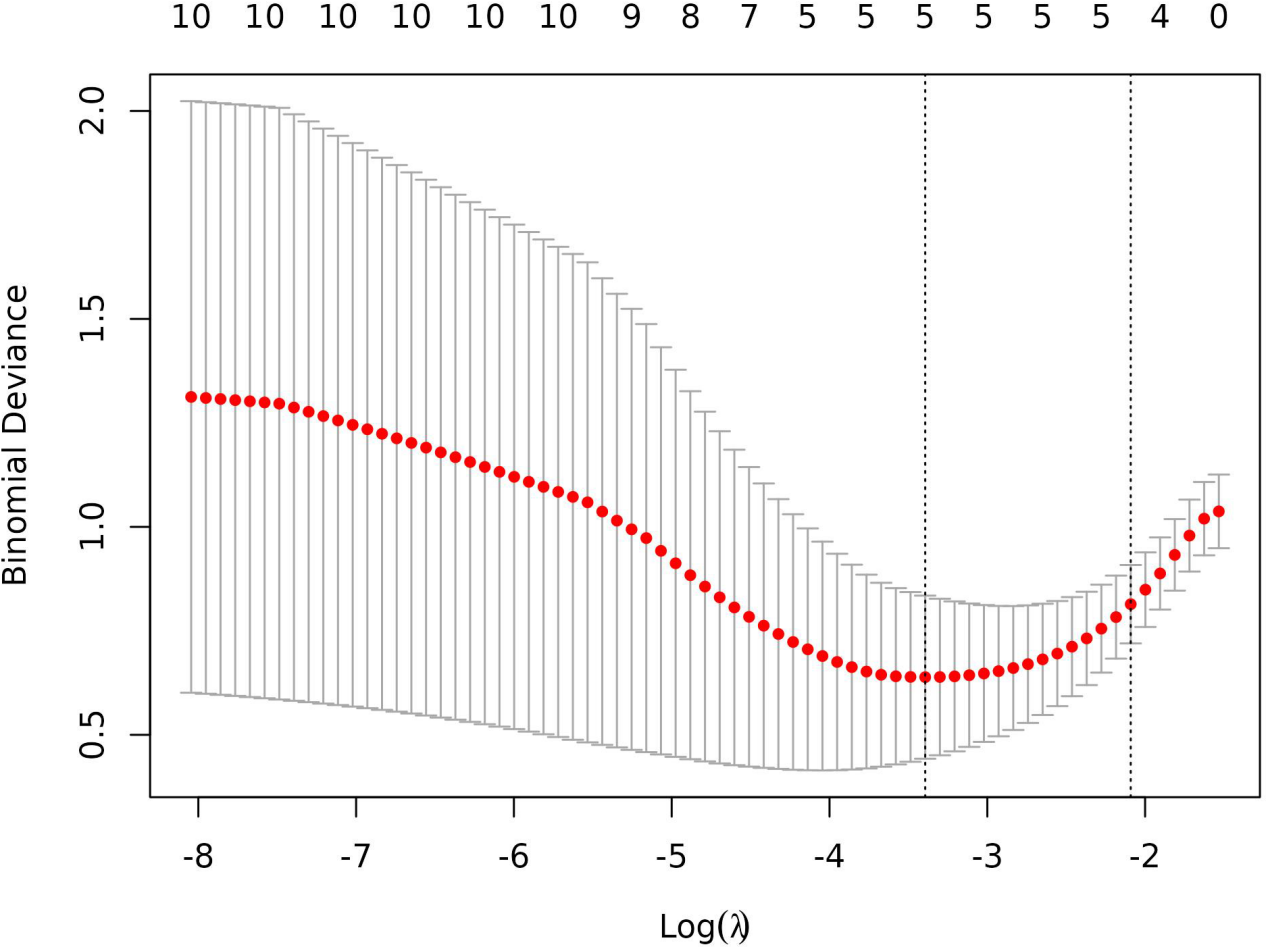
The cross-validation plot of lasso regression.

**Figure 3:**
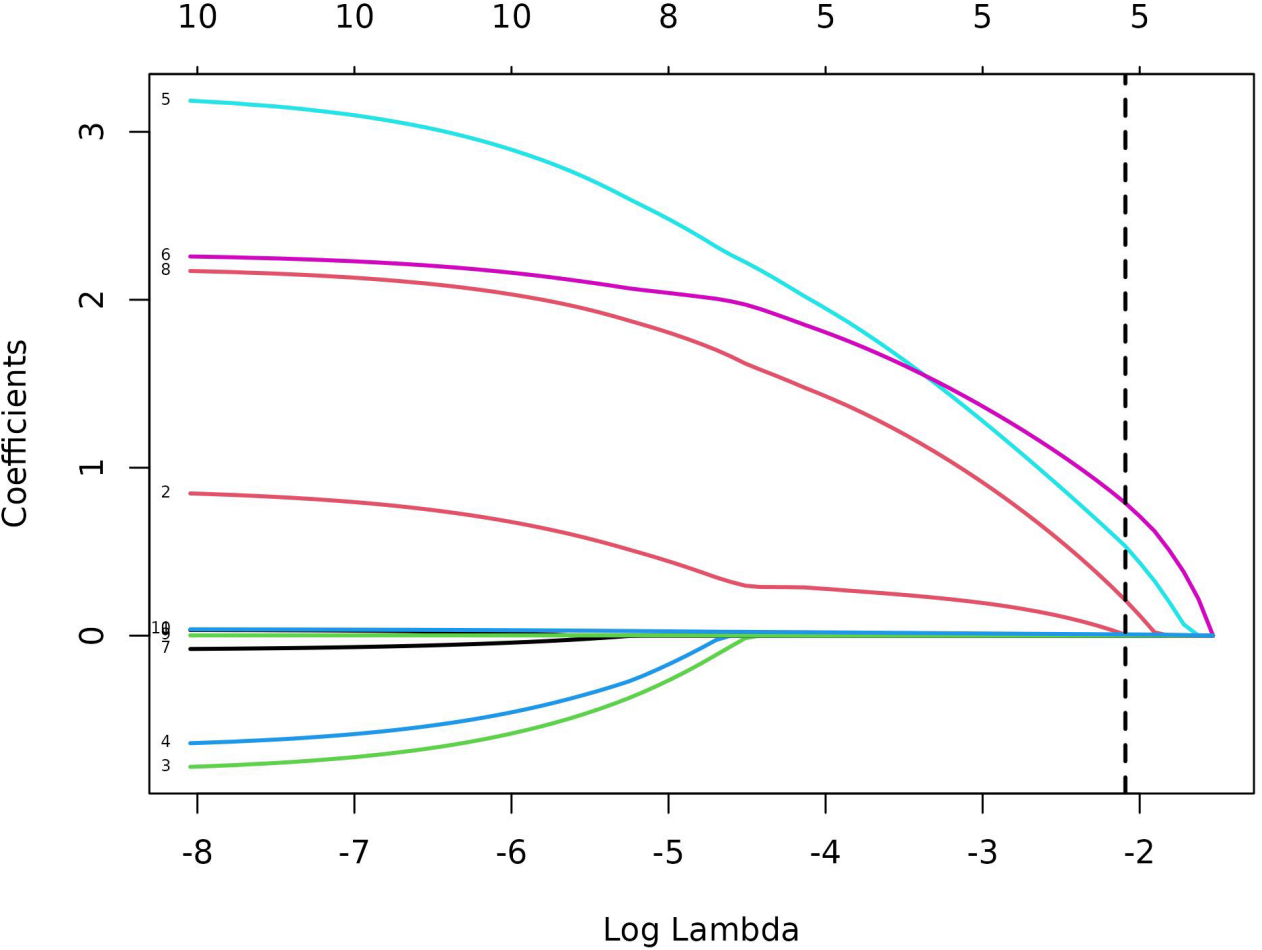
Plot of *λ* vs. nomogram regression coefficient.

In total, 92 children were randomly selected as the training cohort for nomogram construction and 39 children as the internal validation cohort (Table 1). Sex (boy or girl), age (≤2 or >2 years), clinical symptoms (with or without), procedure (LCCE, RALCCE), cyst classification (type I or IVb), thickness of cyst wall (>0.4 or ≤0.4 cm), chronic cholecystitis (with or without), cyst diameter (cm), serum amylase (U/L), and direct bilirubin (μmol/L) were used as independent variables, and the occurrence of postoperative complications was the dependent variable. The selection of variables in the regression nomogram is shown in Figures 2 and 3. Figure 2 describes the relationship between log (*λ*) and the number of independent variables, with the ordinate of binomial deviance, the lower abscissa of log (*λ*), and the upper abscissa of the number of non-zero coefficient independent variables in the nomogram corresponding to different log (*λ*). Line A represents the optimal harmonic coefficient *λ*, *λ*.min = 0.009; line B represents *λ*, for which the simplest nomogram was obtained within one variance range, *λ*.1se = 0.044 (Figure 2). With the increase of *λ*, the estimated coefficients of each independent variable in the Lasso nomogram were compressed, the independent variable coefficients that had little effect on the dependent variable were gradually compressed to 0, and the number of independent variables decreased. Combined with the results of the logistic regression analysis, *λ*.1se = 0.044 was the best selection for the optimal nomogram, while the independent variables included in the Lasso logistic regression nomogram were age (≤2 or >2 years), cyst type (type I or IVb), cyst wall thickness (>0.4 or ≤0.4 cm), with or without of chronic cholecystitis, and direct bilirubin level (μmol/L) (Figure 3).

**Figure 4:**
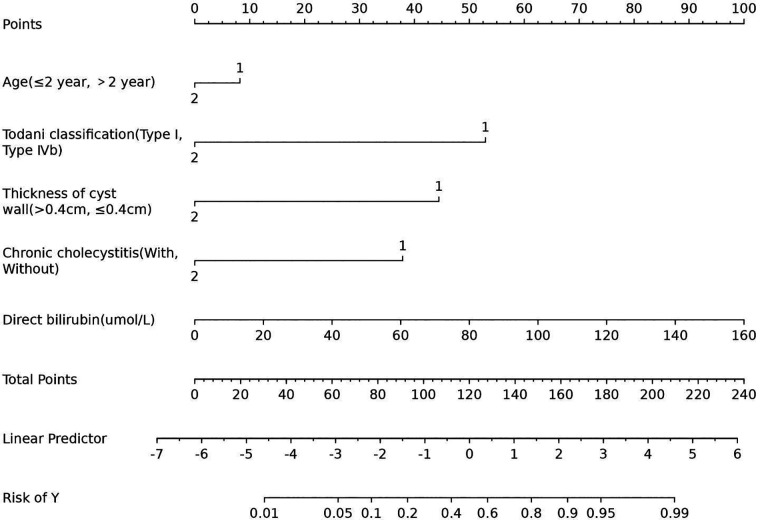
The lasso nomogram for training cohort of children with choledochal cyst.

Upon inclusion, age ≤2 years (OR 1.52; 95% CI 0.16–19.03; *p *= 0.118), Todani classification type I (OR 15.08; 95% CI 1.20–430.87; *p *= 0.054), cyst wall thickness >0.4 cm (OR 9.76; 95% CI 1.83–63.76; *p *= 0.010), with chronic cholecystitis (OR 6.96; 95% CI 1.22–60.93; *p *= 0.043), and direct bilirubin level (OR 1.03; 95% CI 1.00–1.10; *p *= 0.176) were risk factors for postoperative complications of congenital choledochal cysts (Table 3). The Lasso logistic nomogram visually showed the predictive score for the occurrence of postoperative complications in choledochal cysts (Figure 4). The predictive nomogram showed that children aged ≤2 years with choledochal cysts corresponded to 8 points; children with choledochal cyst type I corresponded to 52 points; children with choledochal cyst wall thickening >0.4 cm corresponded to 44 points; children with preoperative cholecystitis corresponded to 38 points; and children with a direct bilirubin level of 160 μmol/L corresponded to 100 points.

**Table 3:** Results of multivariate logistic regression for training cohort.

Characteristic	*N*	Event, *N*	OR	95% CI	*p*-value
Age (years)					
>2	52	2	—	—	
≤2	40	17	1.52	0.16–19.03	0.118
Todani classification					
Type Ⅳb	49	1	—	—	
Type Ⅰ	43	18	15.08	1.20–430.87	0.054
Thickness of cyst wall (cm)					
≤0.4	67	5	—	—	
>0.4	25	14	9.76	1.83–63.76	0.010
Chronic cholecystitis					
Without	50	2	—	—	
With	42	17	6.96	1.22–60.93	0.043
Direct bilirubin (μmol/L)	92	19	1.03	1.00, 1.10	0.176

#### 3.2.2 Parameter estimation and nomogram evaluation

**Figure 5:**
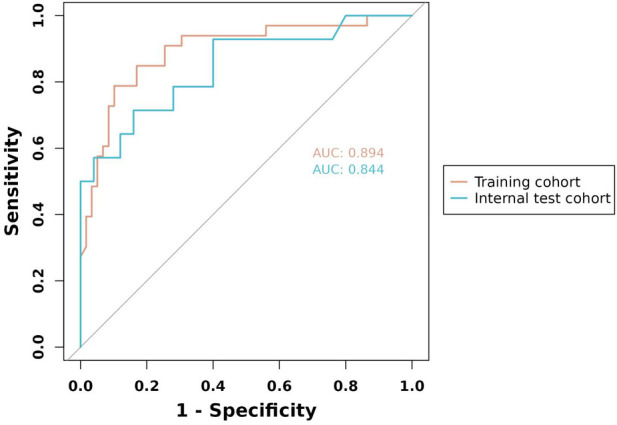
The ROC curve of the training cohort and internal validation cohort.

**Figure 6:**
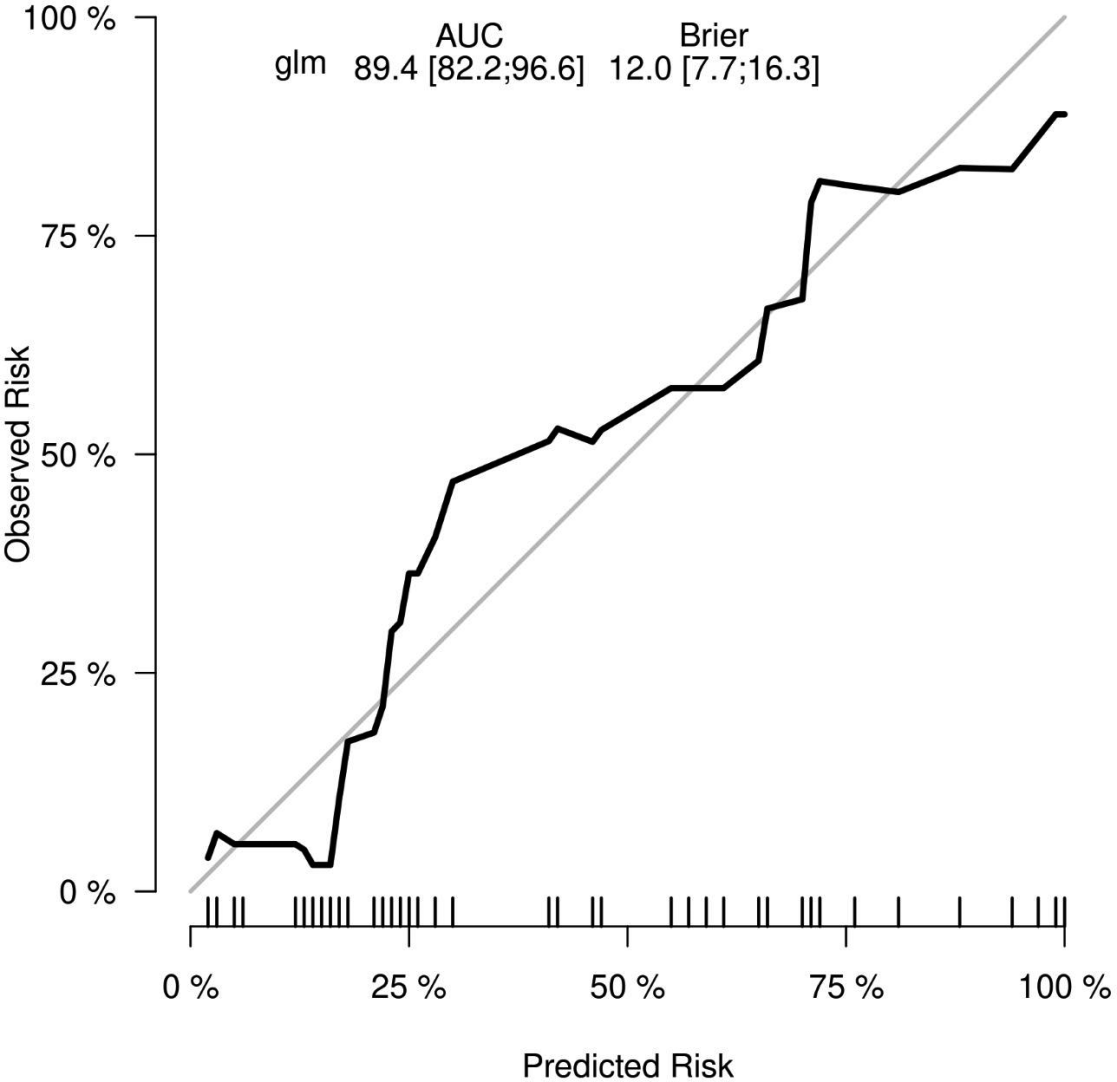
The calibration curve for the lasso nomogram.

**Figure 7:**
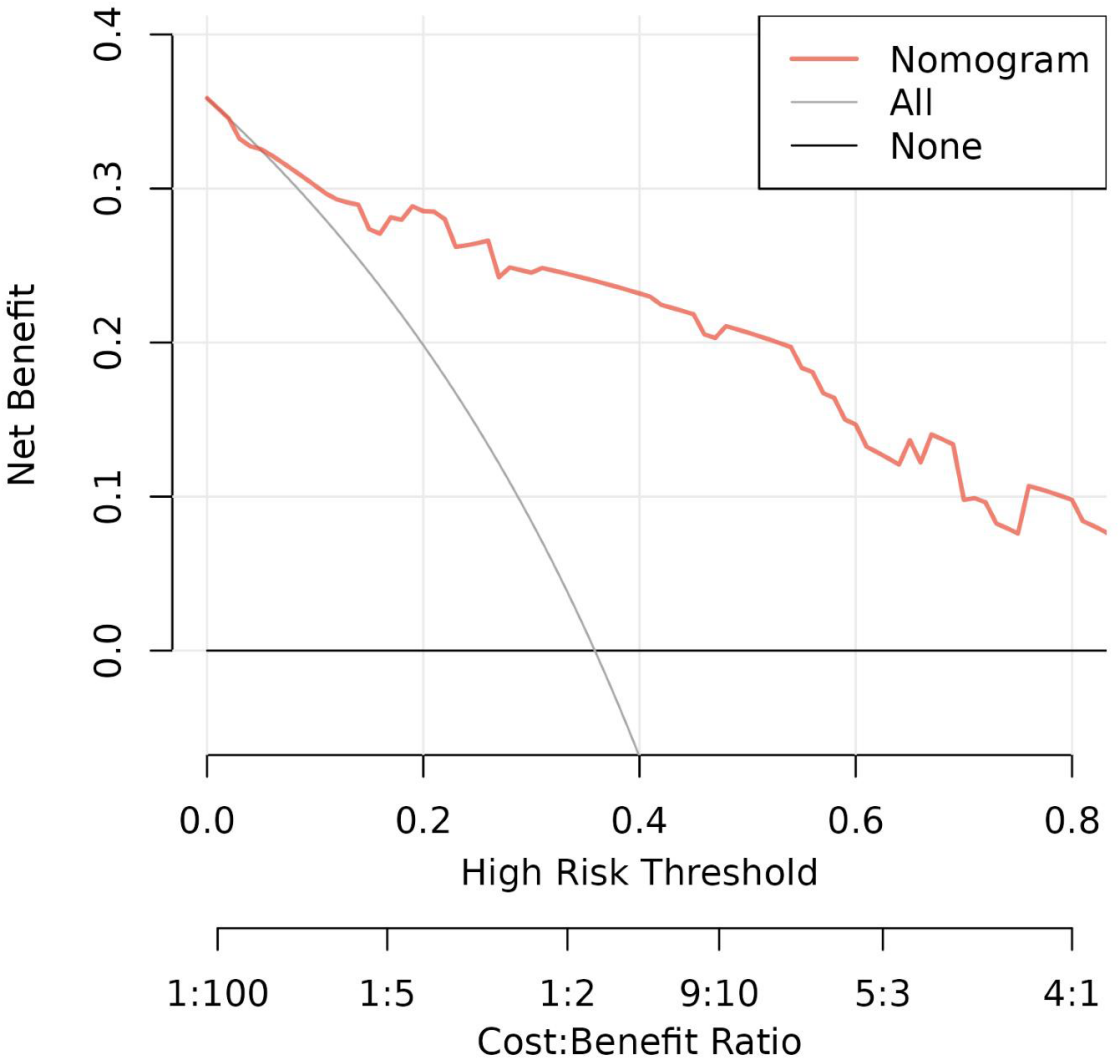
The DCA curve for the lasso nomogram.

To evaluate the discriminant validity and predictive ability of the nomogram, the ROC curves of the training cohort and internal validation cohort were plotted in this study. The ROC curve results showed that the AUC of the nomogram was 0.894 (95% CI 0.822–0.966; *p *< 0.001), the AUC of the internal validation cohort was 0.844 (95% CI 0.804–0.952; *p *< 0.001), Brier = 0.120 (95% CI 0.077–0.163; *p *< 0.001), suggesting that the prediction of the nomogram was more appropriate to the actual risk (Figure 5). On this basis, the slope of the calibration curve for the nomogram was nearly 1, indicating that the prediction of postoperative complications of choledochal cysts was in good agreement with the actual risk of occurrence (Figure 6). At the same time, the decision curve analysis (DCA) curve suggests that the nomogram model has a certain predictive ability (Figure 7). The logistic regression and nomogram were re-analysed, and the new results were presented in Table 1 and Figures 2–7.

## 4 Discussion

The malformation of choledochal cysts is generally thought to be associated with pancreaticobiliary maljunction, in which the regurgitant secretin induces biliary epithelial injury and sphincter dysfunction (23–25), and increases the tendency of biliary fistula and distal obstruction (26, 27). However, the biliary impact of the cyst does not correspond to elevated amylase, but rather high intrabiliary pressure due to the stenosis of the distal CBD and the distal dystopy of the Vater papilla (28, 29). Thus, the Roux-en-Y hepaticojejunostomy is performed to alleviate the biliary stenosis and restore biliary excretion, in which the efficiency is dependent on the characteristics of the cyst and the degree of CBD dilatation. The screening of clinical risk factors is required to design appropriate therapies and ensure successful follow-up care. Here, we filter the perioperative data of children with choledochal cysts to find out the correlative factors and build the prediction nomogram. Upon inclusion, age (≤2 years), cyst type (type I), cyst wall thickness (>0.4 cm), with chronic cholecystitis, and direct bilirubin level (μmol/L) were selected as factors to assess the prognosis of children with choledochal cysts. Both the results of the ROC curve and the calibration curve demonstrate the discriminant validity and predictive ability of the novel nomogram.

The results of the regression analysis suggest that the thickening of choledochal cyst wall is a risk factor for the occurrence of postoperative complications, likely due to the inflammation inducing neovascularization and capillary permeability (30). The pancreaticobiliary maljunction caused by biliary stenosis or malformation induces repeated chronic inflammation, which results in severe adherence to the surrounding tissues and increased operative difficulty. Meanwhile, inflammation precipitates the proliferation of the smooth muscle fibrosis in the cyst wall, leading to further biliary stricture (31). Therefore, the degree of cyst wall thickening can be used to assess the severity of local inflammation, aiding in operative details and prognosis optimization. The elevation of direct bilirubin (>6.8 μmol/L) is also indicative of biliary obstruction and inflammation, and whether there is recurrent stricture of biliary-enteric anastomosis after choledochal cyst surgery. Therefore, the level of direct bilirubin is also included as a risk factor in the prediction nomogram. Moreover, the quality of biliary-enteric anastomosis is related to preoperative chronic cholangitis (32), which is also incorporated into the prediction nomogram. Preoperative chronic cholangitis results in repeated inflammatory stimulation of the CBD and may lead to difficulties during reoperation (33). However, the cyst diameter is not included in the prediction nomogram, even though the detaching of giant cysts (≥6 cm) easily damages the CBD, hepatic duct, and collateral vessels due to dystopia and inflammatory adhesion (34). Intraoperative injury to the CBD with pancreaticobiliary maljunction is more likely to induce postoperative acute pancreatitis (35). The lack of a statistically significant predictive function for cyst diameter may be related to data limitations in our research, warranting further investigation.

The procedure selection for children with choledochal cysts is related to the Todani classification, of which type III is more suitable for undergoing an endoscopic incision and drainage rather than a Roux-en-Y hepaticojejunostomy (36). For type I, which extends to the ductus pancreaticus, or type IVa, with intrahepatic dilatation, the Roux-en-Y jejunostomy needs a further replacement or appending of pancreaticoduodenectomy or hepatectomy during anastomosis reconstruction. Apart from these types, our study suggests that Todani classification type I is a risk factor in children with choledochal cysts. The Roux-en-Y hepaticojejunostomy can effectively improve the intrahepatic dilatation in type IV cysts, thereby avoiding cholangitis caused by biliary stricture with stone (37). Although the type Ic cysts with pancreaticobiliary maljunction more easily lead to the occurrence of postoperative pancreatitis and biliary fistula (25, 27, 38), the type Ic is also mostly associated with patique stenosis (39). In the complication group, there were eight cases of elevated serum amylase (≥150 U/L) after surgery, including four cases of Todani type Ic, two cases of Todani type Ia, and two cases of Todani type IVb cysts. The intraductal protein emboli mostly accompanied with type I cysts causes biliary reflux, also leading to an increased incidence of pancreatitis (25). The protein emboli should be identified and cleaned up during the excision of choledochal cysts (34). The intraductal protein emboli are also likely to be an important factor in improving the prognosis of children. Notably, there is a higher incidence of postoperative biliary strictures for type IV cysts than type I cysts; and the thin smooth muscle of intrahepatic ducts tends to delay anastomotic healing due to the lack of fibrosis (31). Therefore, the correlation between the Todani classification and the occurrence of complications after Roux-en-Y surgery requires comprehensive factors to be considered, so as to develop a more individualized therapy to improve the prognosis of children with choledochal cysts.

The univariate results of the logistic regression analysis show that robot-assisted laparoscopy of choledochal cysts is a protective factor for prognosis compared with laparoscopy. In addition, the predictive nomogram also suggests that age ≤2 years is an important risk factor for children with choledochal cysts, which is not only associated with biliary development in young children, but also the surgical field. Infant choledochal cyst required early surgical intervention, because of not only the predisposition of biliary obstruction, spontaneous perforation, and cirrhosis, but also the growing of cyst infection and adhesions (11, 40). In our opinion, the surgical indications shouldn't be limited as the development of surgical technology, anesthesia and operative nursing. The limited operating space, limited instrument operation, and poor imaging stability weaken the application of laparoscopy, especially in biliary-enteric anastomosis. Meanwhile, the three-dimensional robotic lens imaging system can form more stable and clearer images and increase the accuracy of surgery (13). Robotic surgery is more advantageous in exposing and managing the intrapancreatic portion of choledochal cysts and distal choledochal cysts are easier to detect and free intraoperatively and ligate (41, 42), preventing the infection, stone stricture, and carcinogenesis caused by residual dilated bile ducts. The robot has a flexible and fine simulation manipulator design, can freely bend and rotate in a limited narrow space, and is more accurate when grasping, cutting, suturing, and completing other movements, which is of great significance for hepaticojejunostomy. During the continuous suture of an anastomosis, the electro detaching of biliary-enteric anastomosis and excessive traction of the suture would cause stricture of anastomosis. Inaccurate coaptation of the biliary-enteric mucosa layer during suturing as well as the irritation of tissue from non-absorbable sutures may promote the hyperplasia of the scar around the anastomosis and then stricture. The accessory hepatic duct is more effectively reconstructed through biliary plastic surgery and biliary-enteric anastomosis, with the assistance of robotic arms (43). By reducing the use of scissors to cut the hepatic duct and avoiding the effect of the electrotome, the robotic system can better reduce the risk of bile leakage and stenosis caused by healing of biliary-enteric anastomosis scar, and achieve the discharge requirements earlier. However, the cost of robotic surgery is expensive for children with choledochal cysts. In our research, there are no surgical procedures on the prognosis of children with choledochal cysts. In surgical procedures, the distal port of the CBD is mostly small or atretic to ligature with difficulty, leading to a tendency of pancreatic leakage (44); the exertive detaching of the distal port of the CBD is essential. The diameter of the initial anastomosis should be over 1.0 cm, and an insufficient diameter of the anastomosis is also one of the important causes of postoperative anastomotic stricture (19). Further studies are required to explore the effect of surgical procedures on the prognosis of children with choledochal cyst.

Our research summarized the perioperative data of children with choledochal cysts combined with the postoperative complications in children with choledochal cysts to construct a novel clinical prediction nomogram, providing new assessments for the treatment and prognosis. However, the novel nomogram contains the following limitations: (1) lack of completeness of the data, and the shortage will induce non-extensiveness and inaccuracy of the nomogram; (2) lack of external validation and then authority of the nomogram; (3) the difficulty of operation in children with choledochal cysts closely corresponds to the development of the pancreaticobiliary duct, and the nomogram can be further improved depending on the corresponding data; and (4) factors such as the distance between cystic duct confluence and choledochal cyst, level of transaminase, variation of hepatic artery, and the protein emboli of the hepatobiliary duct can also be included in the clinical prediction nomogram. Above all, the risk factors of postoperative complications of choledochal cysts should be perfected and summarized, so as to construct a stable clinical prediction nomogram related to prognosis, providing reference significance of the prognosis evaluation and treatment for children with choledochal cysts.

## Data Availability

The original contributions presented in the study are included in the article/Supplementary Material, further inquiries can be directed to the corresponding authors.
